# Predicting Response to Systemic Chemotherapy for Advanced Gastric Cancer Using Pre-Treatment Dual-Energy CT Radiomics: A Pilot Study

**DOI:** 10.3389/fonc.2021.740732

**Published:** 2021-09-15

**Authors:** Yi-yang Liu, Huan Zhang, Lan Wang, Shu-shen Lin, Hao Lu, He-jun Liang, Pan Liang, Jun Li, Pei-jie Lv, Jian-bo Gao

**Affiliations:** ^1^Department of Radiology, The First Affiliated Hospital of Zhengzhou University, Zhengzhou, China; ^2^Henan Key Laboratory of Imaging Diagnosis and Treatment for Digestive System Tumor, Zhengzhou, China; ^3^Department of Radiology, Ruijin Hospital, Shanghai Jiao Tong University School of Medicine, Shanghai, China; ^4^Department of DI CT Collaboration, Siemens Healthineers Ltd, Shanghai, China; ^5^Department of Oncology, The First Affiliated Hospital of Zhengzhou University, Zhengzhou, China

**Keywords:** dual-energy CT, radiomics, response prediction, systemic chemotherapy, gastric cancer

## Abstract

**Objective:**

To build and assess a pre-treatment dual-energy CT-based clinical-radiomics nomogram for the individualized prediction of clinical response to systemic chemotherapy in advanced gastric cancer (AGC).

**Methods:**

A total of 69 pathologically confirmed AGC patients who underwent dual-energy CT before systemic chemotherapy were enrolled from two centers in this retrospective study. Treatment response was determined with follow-up CT according to the RECIST standard. Quantitative radiomics metrics of the primary lesion were extracted from three sets of monochromatic images (40, 70, and 100 keV) at venous phase. Univariate analysis and least absolute shrinkage and selection operator (LASSO) were used to select the most relevant radiomics features. Multivariable logistic regression was performed to establish a clinical model, three monochromatic radiomics models, and a combined multi-energy model. ROC analysis and DeLong test were used to evaluate and compare the predictive performance among models. A clinical-radiomics nomogram was developed; moreover, its discrimination, calibration, and clinical usefulness were assessed.

**Result:**

Among the included patients, 24 responded to the systemic chemotherapy. Clinical stage and the iodine concentration (IC) of the tumor were significant clinical predictors of chemotherapy response (all *p* < 0.05). The multi-energy radiomics model showed a higher predictive capability (AUC = 0.914) than two monochromatic radiomics models and the clinical model (AUC: 40 keV = 0.747, 70 keV = 0.793, clinical = 0.775); however, the predictive accuracy of the 100-keV model (AUC: 0.881) was not statistically different (*p* = 0.221). The clinical-radiomics nomogram integrating the multi-energy radiomics signature with IC value and clinical stage showed good calibration and discrimination with an AUC of 0.934. Decision curve analysis proved the clinical usefulness of the nomogram and multi-energy radiomics model.

**Conclusion:**

The pre-treatment DECT-based clinical-radiomics nomogram showed good performance in predicting clinical response to systemic chemotherapy in AGC, which may contribute to clinical decision-making and improving patient survival.

## Introduction

Gastric cancer (GC) remains one of the most common malignant tumors in the world, and its morbidity and mortality rank fifth and third, respectively. There were more than a million new cases and an approximated 784,000 deaths worldwide in 2018 (1); moreover, most GC cases are diagnosed at an advanced stage (2). It is therefore essential to select an effective treatment regimen for advanced gastric cancer (AGC) to maximize the overall therapeutic benefits. Chemotherapy can improve survival and quality of life for patients present with unresectable, locally advanced, or metastatic GC (3). Furthermore, the overall survival of AGC patients who are treated with systemic chemotherapy was 8 months longer than with optimal supportive care alone (4–8). However, tumor response rate of most treatment regimens is less than 40% and chemotherapy drugs can cause serious side effects in some patients (9, 10). Hence, pre-treatment prediction of tumor response to systemic chemotherapy may translate into more precise patient selection and individualized medicine, which are of great clinical significance.

Dual-energy CT (DECT) is a milestone imaging tool that generates a rich amount of DECT quantitative information. The virtual monochromatic images (VMI) derived from DECT have been used in the diagnosis and prediction of tumors, including classification of parotid neoplasms, the evaluation and characterization of cervical lymphadenopathy, prediction of lymph node metastasis in GC, and classification of clear cell renal cell carcinoma (11–15). In terms of predicting the treatment efficacy, Tang et al. demonstrated that iodine concentration (IC) on DECT could evaluate efficacy response of GC to neoadjuvant chemotherapy (16). However, to our knowledge, the application and potential advantages of multi-energy virtual monochromatic image datasets in predicting therapeutic response of GC have not been explored. Theoretically, there is a rich amount of quantitative information in the variation of energy-dependent attenuation in different tissues. Given the dynamic and heterogeneous nature of tumor (17, 18), performing radiomics analysis on monochromatic images may improve the predictive capabilities (11).

Radiomics can noninvasively analyze tumor biology, distinguish the subtle differences that human eyes cannot discern, quantify tumor heterogeneity, and monitor tumor development and response to treatment (19–23). Through extensive extraction of quantitative features, radiomics can delineate tumor heterogeneity metrics, which may reflect pathophysiological characteristics associated with treatment response (23–25). In fact, pre-treatment radiomics with other CT techniques has been proven to non-invasively predict treatment responses of GC (26–29).

Therefore, we aimed to establish and assess a clinical-radiomics nomogram from pre-treatment DECT scans to predict clinical response to systemic chemotherapy in patients with AGC, and to verify whether radiomics performed on multi-energy VMI datasets is more helpful in predicting response.

## Materials and Methods

### Patients

This multi-center, retrospective study was approved by the institutional review board, and the requirement for informed consent was waived due to the retrospective study design. A total of 69 consecutive patients from two independent institutions (49 from the Zhengzhou University First Affiliated Hospital between March 2014 and November 2019 and 20 from Shanghai Jiao Tong University Ruijin Hospital between November 2017 and February 2019) were collected. The inclusion criteria were as follows: (1) histologically confirmed primary gastric adenocarcinoma; (2) no prior history of radiotherapy, chemotherapy, or other treatments that might affect the blood supply to the tumor; (3) no serious heart and renal insufficiency and other important viscera lesions; (4) received baseline contrast-enhanced DECT examinations within 1 week before chemotherapy; (5) treated with systemic chemotherapy due to metastatic, unresectable, and recurrent GC or tumors surrounding major vessels on CT examination (cT4a~bNxM0~1); (6) ECOG PS 0–2. The exclusion criteria were as follows: (1) patients with co-malignancy; (2) incomplete clinical data at baseline; (3) motion artifacts on CT; (4) lesions with cystic changes or cavitation; and (5) intolerance to chemotherapy. Baseline clinicopathological data, including age, sex, and clinical stage, were obtained from retrospective electronic records.

### Systemic Chemotherapy Regimen and Treatment Response Evaluation

In our study, enrolled patients were mainly treated with capecitabine plus oxaliplatin (XELOX) regimen or S-1 plus oxaliplatin (SOX) regimen.

In detail, patients were given capecitabine at a dose of 1,000 mg/m^2^ (or S-1: 60 mg/m^2^) orally twice daily from day 1 to day 14. Furthermore, oxaliplatin (130 mg/m^2^) was given intravenously for 2 h on day 1. Cycles were repeated every 21 days, and the toxicity of chemotherapy was evaluated after each cycle. At least six cycles of treatment were given unless there was disease exacerbation, unacceptable toxicity, or death occurred.

### Evaluation of Treatment Response

Post-treatment CT images were obtained within 3 weeks after completion of chemotherapy. The treatment response was assessed by the change of the sum of the maximum diameters for all target lesions in the pre- and post-chemotherapy CT images.

**Figure 1:**
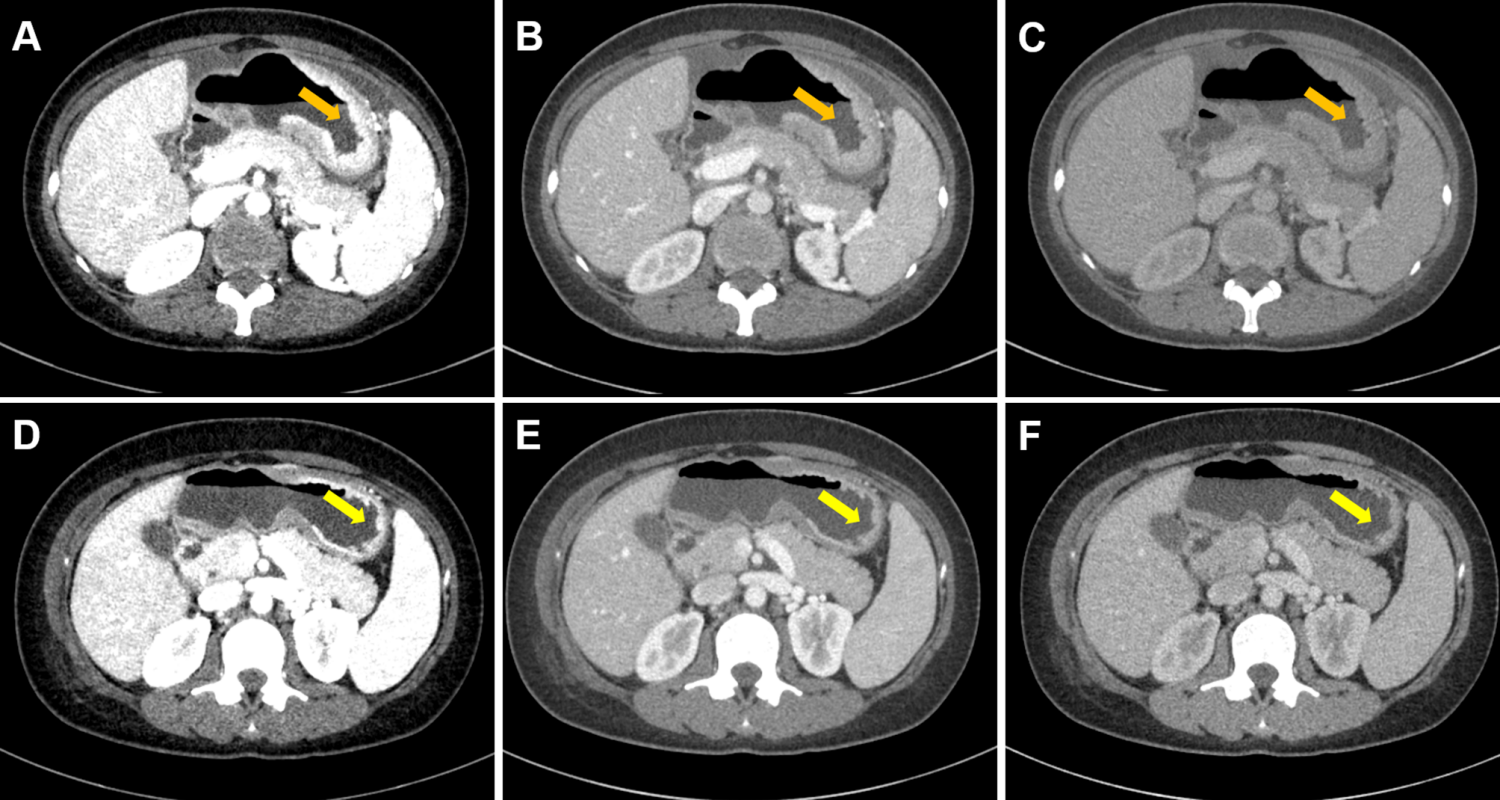
Case 1. Portal phase DECT images of a 34-year-old female with GC patient who responded to chemotherapy. **(A–C)** Diffuse thickened gastric wall was seen at the gastric body (arrow). **(D–F)** Slightly thickened gastric wall was seen at the gastric body, and the lesion was significantly regressed (arrow). **(A–C)** were monochromatic images of 40, 70, and 100 keV before chemotherapy, respectively. **(D–F)** were monochromatic images of 40, 70, and 100 keV after chemotherapy, respectively.

**Figure 2:**
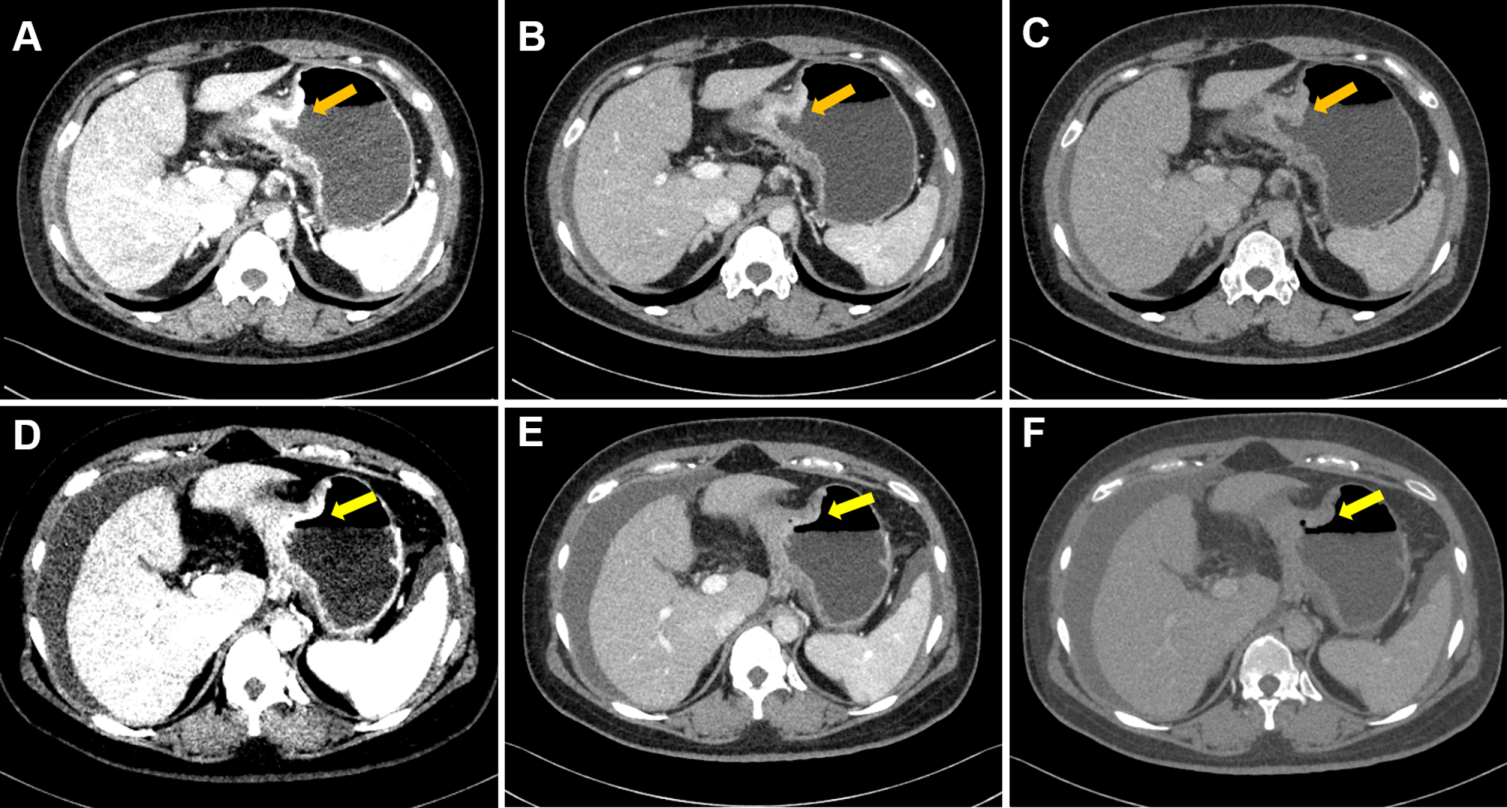
Case 2. Portal phase DECT images of a 40-year-old female patient with GC who did not respond to chemotherapy. **(A–C)** An irregular wall-thickening lesion at the gastric body was present (arrow). **(D–F)** There was no obvious change (slightly regression) in the lesions of gastric body (arrow). **(A–C)** were monochromatic images of 40, 70, and 100 keV before chemotherapy, respectively; **(D–F)** were monochromatic images of 40, 70, and 100 keV after chemotherapy, respectively. Note: After completion of chemotherapy, the patient was diagnosed with aggravated peritoneal metastasis.

The short-term therapeutic response was evaluated with the standard of Response Evaluation Criteria in Solid Tumors (RECIST v. 1.1) (30). Based on current study purpose, we classified patients with complete response (CR, complete disappearance of all target lesions and no new lesions) or partial response (PR, a reduction ≥30% in the sum of the diameters of target lesions) as responders, while others with stable disease (SD, neither partial response nor progressive disease) or progressive disease (PD, a ≥20% size increase or new disease) were classified as non-responders (**Figures 1** and **2**).

### CT Image Acquisition

All patients fasted for 8 to 12 h before examination and took 800–1000 ml of warm water before the CT scan, where the patients were placed in a supine position with head first and breath-hold.

CT scans were performed using multi-vendor Dual-Energy CT (Discovery CT750 HD scanner, GE Healthcare, Milwaukee, WI, USA; SOMATOM Force scanner, Siemens Healthineers, Forchheim, Germany). The patients underwent contrast-enhanced DECT scans, including the arterial phase (AP) and venous phase (VP). After unenhanced CT was performed, the main contrast agent (Ultravist 370, Bayer Schering Pharma, Thüringen. Germany) was infused intravenously through the antecubital vein at a rate of 3.0 ml/s (1.5 ml/kg) using a pump injector. AP and VP contrast-enhanced CT images were achieved after a post-injection delay of 30 and 70 s, respectively. The scanning parameters were summarized as follows (1) Discovery CT750 HD: using fast tube voltage switching between 80 and 140 kVp, tube current: 375 mA, pitch: 1.375:1, rotation time: 0.6 s, detector width: 40 mm, collimation: 128*0.6 mm, FOV: 400 × 400 mm; reconstruction algorithm: STAND; reconstructed section thickness:1.25 mm slice thickness: 5 mm. (2) SOMATOM Force: tube voltage: 100/Sn150 kVp; effective tube current-time product: 200/125 mAs; FOV: 374 × 374 mm; rotation time: 0.5 s; pitch: 0.6; reconstructed section thickness: 1.25 mm slice thickness: 5 mm; kernel: Qr40; collimation: 128*0.6 mm.

### Image Analysis

The CT images were transferred to dedicated workstations with dual-energy software (Syngo.via, Version VB10, Siemens Healthineers, Forchheim, Germany; ADW 4.7, GE Healthcare, Milwaukee, WI, USA).

A 15-year experienced gastrointestinal radiologist interpreted the dual-energy images with the knowledge that all patients had GC confirmed by endoscopic biopsy. Clinical lymph node staging (cN) and distant metastasis staging (cM) were evaluated according to the 8th edition of AJCC guidelines (31), and the distant metastatic sites were recorded. The maximal thickness (the largest short diameter perpendicular to the longest axis on the maximal cross-section) of the primary tumor was measured. The Borrmann classification of the tumor was also assessed (32). A free-hand, VP-based individualized region of interest (ROI) was manually delineated on iodine-based material decomposition images in the largest cross-sectional area by the reader, and then the ICs (mean value, units of 100 μg/ml) of the lesion in the ROI was recorded. Meanwhile, circular ROIs were carefully placed at the same slice to avoid calcified plaques and subsequently obtain the aortic ICs. Finally, the iodine ratio of the lesion to aorta was taken as normalized iodine concentration (NIC = IC lesion/IC aorta).

### Tumor Segmentation and Feature Extraction

We conducted lesion segmentation and radiomics feature extraction with a prototypical software (Syngo Frontier, Radiomics 1.0.9a, Siemens Healthineers, Germany). Venous phase images were previously reported as the best phase for GC visualization (14, 27, 33) and therefore were used for tumor segmentation. In order to seize the energy-dependent changes in tissue attenuation, we selected monochromatic images of 40, 70, and 100 keV as typical dual-energy datasets for feature extraction. The volumes of interest (VOI), referred to whole tumor regions in three dimensions on venous phase contrast-enhanced DECT images, was delineated by a radiologist with 7 years of experience and reviewed by a radiologist with 10 years of experience to minimize possible bias (**Supplemental Appendix 1; Figure S1**). The software provides a variety of options to customize image pre-processing before radiomic feature extraction, including wavelet filtering, Laplacian of Gaussian filtering, and non-linear intensity transforms including logarithm, exponential, square, and square root operations. The extracted features were reproducible and matched the benchmarks of image biomarker standardization initiative (IBSI) (34).

Finally, 1691 radiomics features were extracted from each patient in each single-energy image set, including 17 shape features, 324 first-order features, and 1,350 texture features **Supplemental Appendix 2; Table A1**.

### Feature Selection and Radiomics Model Establishment

To prevent overfitting or selection bias in our radiomics model, univariate logistic regression analysis (*p* < 0.05) and LASSO regression were used to screen out the most relevant informative radiomic features of chemotherapy response. Tenfold cross-validation was performed to determine the optimal value of regularization parameter λ at minimum MSE. Based on the selected features, the radiomics model was established by multivariate logistic regression algorithm. Three single-energy (40-keV, 70-keV, and 100-keV) radiomics models and a multi-energy (combined three single-energy features) radiomics model were established. The process of LASSO is shown in **Supplemental Appendix 3; Figure S2**.

### Clinical Model and Nomogram Establishment

Univariate and multivariate logistic regression analysis were used to determine the independent clinical predictors related to chemotherapy response.

The candidate factors of univariate logistic regression analysis included age, gender, clinical stage, cN stage, cM stage, distant metastatic sites, location, Borrmann classification, thickness, and IC and NIC value. Odds ratio and 95% confidence interval (CI) were calculated. The significant variables (*p*-value < 0.05) in the univariable analysis were considered in the multivariate logistic regression analysis. Then, the independent clinical predictive factors were determined and the clinical model was established. In addition, a combination model (ComModel) was established by combining the selected clinical predictor with multi-energy radiomics model to explore the added value of the additional dual-energy information. Meanwhile, the ComModel was visualized as a nomogram to predict individualized probability of response.

### Evaluation and Comparison of Model Performance

**Figure 3:**
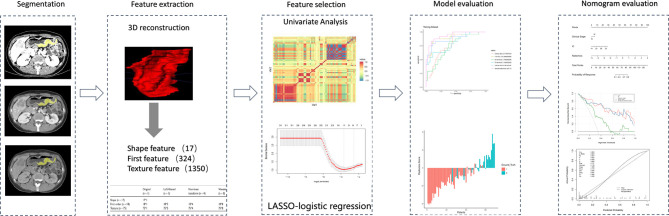
Radiomics workflow of the present study. The radiomics procedure consists mainly of five steps: volume of interest segmentation, feature extraction, feature selection, model evaluation, and nomogram evaluation.

Evaluation of the model contained discrimination, calibration, and clinical usefulness. The receiver operating characteristic (ROC) curve analysis was used to evaluate the discrimination performance of each model, while the DeLong test was used to compare the differences in area under the curve (AUC) among different models. Calibration curves were carried out to describe calibration performance according to agreement between predicted and actual probability of response. Decision curve analysis (DCA) was employed to estimate the clinical usefulness of the model based on the net benefit at different threshold probabilities. The radiomics flowchart of our study is shown in **Figure 3**.

### Statistical Analysis

Feature selection, model construction, and performance evaluation were performed on R software package (version 3.6.3). Other statistical analyses were conducted with SPSS25.0 software (IBM, USA). A two-tailed *p*-value<0.05 was considered statistically significant.

Normality of distribution of continuous variables was tested using a Kolmogorov–Smirnov test. The differences in continuous variables were assessed by using analysis of variance (ANOVA), and categoric variables were compared using the χ^2^ test.

## Results

### Clinical Characteristics

The general demographic characteristics, clinicopathological characteristics, and dual-energy parameters of the patients are shown in **Supplemental Appendix 4; Table A2**. A total of 69 (median age 56 years, range 23–84 years) patients with AGC were analyzed in this study. The number of AGC patients with stage IV disease was 47 (68.1%). There were 10 patients (14.5%) presented with diffuse lesions (lesion location ≥2). Fifty-eight patients (84.1%) demonstrated evidence of lymph node involvement. Distant metastases were found in 40 patients (58.0) and 14 of them (20.3%) presented with liver metastasis.

According to the results of response assessment, patients were divided into responder (*n* = 24) and non-responder (*n* = 45) groups. Baseline characteristics of the two groups are summarized in **Table 1**. Clinical Stage, Borrmann classification, and IC were found to be significantly different between groups. Furthermore, univariate and multivariate logistic regression analyses demonstrated that clinical stage and IC value were independent clinical predictors of response to chemotherapy for AGC (**Table 2**).

**Table 1:** Baseline characteristics of responder and non-responder groups.

Characteristics	Responder (*n* = 24)	Non-responder (*n* = 45)	*p*
Age (years)	57.25 ± 12.44	53.22 ± 14.10	0.250
Sex			0.352
Female	9	12	
Male	15	33	
ECOG			0.136
PS 0	11	29	
PS 1–2	13	16	
Clinical Stage			0.018*
III	12	10	
IV	12	35	
cN stage			0.768
N0	5	6	
N1	10	17	
N2	5	11	
N3	4	11	
Metastatic sites			0.597
Absent	12	17	
Liver	4	10	
Lung	0	2	
Other^a^	8	16	
Location			0.879
Upper	11	19	
Middle	3	9	
Lower	6	11	
Diffuse	4	6	
Borrmann type			<0.001*
I–II	2	8	
III	20	15	
IV	2	22	
Thickness (cm)	2.165 ± 0.723	2.520 ± 0.855	0.093
IC (100 μg/ml)	24.857 ± 3.153	21.780 ± 3.379	0.001*
NIC	0.078 ± 0.044	0.127 ± 0.067	0.257

*p-value < 0.05. Data (%) are the proportion of sample size or mean value ± SD.

a peritoneum, distant lymph node, adrenal gland, ovary; N, lymph node; IC, iodine concentration; NIC, normalized iodine concentration.

**Table 2:** Clinical predictors for response to chemotherapy in patients with AGC.

Characteristic	Univariable analysis	*p*-value	Multivariable analysis	*p*-value
	OR (95% CI)		OR (95% CI)	
Age (years)	1.023 (0.984–1.062)	0.249		
Sex		0.354		
Male	Reference			
Female	1.650 (0.573–4.753)			
ECOG		0.139		
PS 0	Reference			
PS 1–2	2.142 (0.781–5.873)			
Clinical stage		0.021^*^		0.029^*^
III	Reference		Reference	
IV	0.286 (0.098–0.829)		0.251 (0.072–0.869)	
cN stage				
N0	Reference	–		
N1	0.706 (0.170–2.923)	0.631		
N2	0.545 (0.111–2.673)	0.455		
N3	0.436 (0.084–2.269)	0.324		
cM stage		0.329		
M0	Reference			
M1	0.607 (0.223–1.653)			
Location				
Upper	Reference	–		
Middle	0.576 (0.128–2.588)	0.472		
Lower	0.942 (0.272–3.260)	0.925		
Diffuse	1.152 (0.266–4.993)	0.850		
Borrmann type				
I–II	Reference	–		
III	4.500 (0.806–25.122)	0.086		
IV	0.350 (0.041–2.977)	0.336		
Thickness (cm)	0.551 (0.272–1.119)	0.099		
IC (100 μg/ml)	1.334 (1.108–1.605)	0.002^*^	1.309 (1.067–1.605)	0.010^*^
NIC	6.950 (0.248–194.682)	0.254	4.373 (0.077–247.896)	0.474

*p-value < 0.05. CI, confidence interval; OR, odds ratio; N, lymph node; M, distant metastasis; IC, iodine concentration; NIC, normalized iodine concentration.

### Radiomics Feature Selection

Based on LASSO regression, we obtained 8, 4, 6, and 11 most significant radiomics features with non-zero coefficients as the predictive radiomics features from the 40-keV, 70-keV, 100-keV, and multi-energy groups, respectively. The distribution of the selected radiomics features of the corresponding model coefficients is shown in **Supplemental Appendix 5**, **Tables A3** and **A4**.

### Evaluation and Comparison of Model Performance

**Figure 4:**
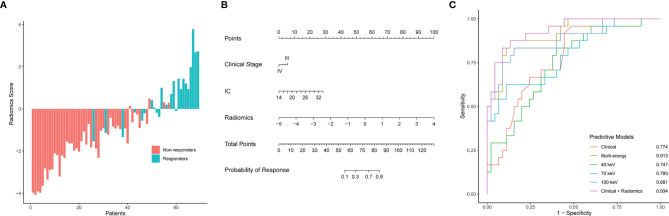
Radiomics score, nomogram developed with the combined model, ROC curve analysis of all models. **(A)** Waterfall plot for distribution of radiomics scores for each patient. **(B)** The developed clinical-radiomics nomogram to predict response to chemotherapy in patients with gastric cancer. **(C)** ROC curves of all models for predicting response to chemotherapy. *AUC, area under the curve; Clinical-radiomics*, nomogram.

Radiomics model: The 100 keV radiomics model had the better predictive value among the three monochromatic radiomics models, with an AUC of 0.881 (95% CI 0.791–0.971). The AUC was 0.747 (95% CI: 0.628–0.866) for the 40-keV radiomics model and 0.793 (95% CI 0.678–0.908) for the 70-keV radiomics model. The AUC of the multi-energy radiomics model was 0.914 (95% CI 0.846–0.982) (**Figure 4A**).

Clinical model: Clinical stage and IC were included in the model. The AUC of the clinical model was 0.774 (95% CI 0.628–0.866).

Combined model: A combined clinical-radiomics model (ComModel) was established incorporating multi-energy radiomics features, clinical stage, and IC value while presented as a nomogram (**Figure 4B**). The AUC of ComModel was 0.934 (0.877–0.991).

The AUC of the multi-energy radiomics model predicting response probability was superior to two monochromatic radiomics models and the clinical model. The ComModel achieved best discrimination among all models with an AUC of 0.934. Besides, there was no significant difference between ComModel, the 100-keV model, and the multi-energy model (*p* = 0.138 between the multi-energy model and ComModel, *p* = 0.073 between ComModel and the 100-keV model, *p* = 0.221 between the 100-keV model and the multi-energy model). ROC curves and detailed performances of the six models are illustrated in **Figure 4C** and **Table 3**. A comparison of discrimination of these models is demonstrated in **Table 4**.

**Table 3:** Radiomics, clinical-only, and clinical-radiomics model predictive performance.

Model	AUC (95% CI)	SPE (%)	SEN (%)	ACC (%)	PPV (%)	NPV (%)
40 keV	0.747 (0.628–0.866)	60.0	83.3	68.1	52.6	87.1
70 keV	0.793 (0.678–0.908)	88.9	62.5	79.7	75.0	81.6
100 keV	0.881 (0.791–0.971)	84.4	83.3	84.1	74.1	90.5
Full	0.914 (0.846–0.982)	86.6	87.5	86.9	77.7	92.8
Clinical	0.775 (0.665–0.884)	55.6	91.7	68.1	52.4	92.6
ComModel	0.934 (0.877–0.991)	91.1	83.3	88.4	83.3	91.1

AUC, area under the curve; SPE, specificity; SEN, sensitivity; ACC, accuracy; PPV, positive predictive value; NPV, negative predictive value; Full, multi-energy; ComModel, Clinical-Radiomics; CI, confidence interval.

**Table 4:** Comparison of discrimination of all models.

**Model 5**	0.17				
**Model 4**	<0.01*	0.02*			
**Model 3**	0.10	0.22	0.11		
**Model 2**	<0.01*	0.01*	0.81	0.17	
**Model 1**	<0.01*	<0.01*	0.74	0.02*	0.52
	**Model 6**	**Model 5**	**Model 4**	**Model 3**	**Model 2**

*p < 0.05. Model (1) corresponds to the model based on selected 40-keV radiomics features, model (2) corresponds to the model based on selected 70-keV radiomics features, model (3) corresponds to the model based on selected 100-keV radiomics features, model (4) corresponds to the clinical model, model (5) corresponds to the model based on selected multi-energy radiomics features, and model (6) corresponds to the model combining multi-energy radiomics features and clinical features.

### Evaluation of Clinical-Radiomics Nomogram Performance

**Figure 5:**
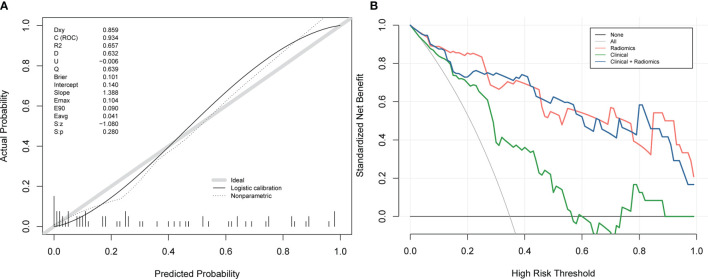
Calibration curves and decision curve analysis of the nomogram. **(A)** Calibration curves of the nomogram. The *x*-axis and the *y*-axis show the nomogram-predicted response probabilities and the actual probabilities, respectively. The calibration curve presents the calibration of the nomogram in terms of the agreement between the predicted response to chemotherapy and the observed probabilities of response to chemotherapy. The diagonal gray line presents a perfect prediction, and the black solid line presents the predictive performance of the nomogram. Better prediction is indicated by a closer fit of the black solid line to the diagonal gray line. **(B)** Decision curve analysis for the combined model, multi-energy radiomics model, and clinical model. The *y*-axis represents the net benefit. The gray line represents the assumption that all patients have a response to chemotherapy; however, the black line is the opposite. The blue line represents the combined model. The red line represents the multi-energy radiomics model. The green line represents the clinical model.

The calibration curves of the nomogram (**Figure 5A**) showed a good fit between predictive probability of response and actual response rate. Non-significant statistics of the accompanied Hosmer–Lemeshow test (*p* = 0.280) implied that the nomogram was adequately calibrated without departure from the ideal fit. The decision curve analysis (**Figure 5B**) demonstrated good performance of the multi-energy radiomics model and the nomogram in terms of clinical decision-making, which added more benefits than either a treat-all or treat-none scheme. In addition, the analysis showed that the nomogram and multi-energy radiomics model had a similar clinical application value, and their prediction performance was better than that of the clinical model.

## Discussion

In this study, we built a DECT-based clinical-radiomics nomogram for systemic chemotherapy response prediction in AGC using datasets from two centers. The constructed nomogram, which combined clinical stages, IC, and DECT-derived radiomics features, demonstrated satisfactory discriminative ability, and can be used to stratify patients who are more likely to benefit from systemic chemotherapy. Furthermore, our study demonstrates that radiomics features extracted from the virtual monochromatic images can reflect heterogeneity of gastric cancer and that radiomics may serve as a promising technique for predicting the response to treatment in patients with AGC.

Existing radiomics models for predicting response to systemic chemotherapy used both pre-treatment and post-treatment CT images (35). However, the post-treatment nature could narrow its extensive utility in clinical therapy decision-making (36). Thus, pre-treatment images were selected to construct prediction models in the current study. Using a pre-treatment predictive model, clinicians can identify the chemosensitivity of patients, thereby better stratifying patients for more appropriate treatment regimens (36). As a result, the pre-treatment predictive model may broaden its application in the clinical settings and help personalize treatment and improve prognosis of AGC patients.

Dual-energy imaging extends the capabilities of conventional CT offering potentials to improve lesion detection and characterization (33). At present, some scholars have been committed to the combination of dual-energy CT and radiomics or texture analysis (13, 37–42). However, most feature extractions were based on single-energy monochromatic images, 120 kV equivalent mixed images, or iodine images. There were few studies on feature extraction based on multi-energy images, especially in gastric cancer. Li et al. (14) found that the multi-energy image-based radiomics model could better predict lymph node metastasis (LNM) for gastric cancer when compared to the clinical and single-energy model. In terms of monoenergetic selection for radiomics model construction, 70 keV was used as it could resemble a 120-kVp conventional single-energy CT acquisition, while having a higher contrast-to-noise ratio and less image noise (43–46). Meanwhile, according to basic CT physics and algorithms, we also selected 40-keV images as the representative of low-energy dataset (40–70 keV) to reflect the tissue enhancement characteristics and 100-keV images as the representative of high-energy dataset (100–140 keV) to reflect the tissue non-enhanced characteristics. In our comparison of three monoenergetic radiomics models, the 100-keV model achieved a better performance. High-energy monochromatic images have higher image quality and lower background parenchymal noise (47, 48). Thus, we speculate that the radiomics features based on low-noise, high-energy images reflecting the tissue non-enhanced nature are more likely to seize the heterogeneity of tumors. Notably, the 100-keV model did not significantly differ from the multi-energy model or the clinical-radiomics nomogram in terms of response prediction. This finding was consistent with a previous study that the potential benefits of multi-energy images must be evaluated on a case-by-case basis (13). From this, the 100-keV images not only was visually comfortable and extensively useful in clinical routine display (14), but also showed good performance in predicting systemic chemotherapy response of AGC.

DECT-derived IC represents iodine deposition in tissues and is deemed as an alternative measure for tumor vascularity and perfusion (49). Previous studies have explored the application of IC in the field of oncology for diagnosis, the prediction of lymph node metastasis, and the evaluation of therapy response (50–52). Tang et al. revealed that the tumor IC was in good agreement with the pathological regression in evaluating the response of GC to neoadjuvant chemotherapy, and prediction efficacy of IC was superior to that of tumor thickness (16). In the present study, univariate and multivariate analysis results showed that IC was an independent predictor of the response of chemotherapy for GC. Moreover, the IC value of the non-response group was significantly lower than that of the response group, which may indicate that the relatively low blood supply of the tumor before chemotherapy has some difficulties in the targeted organ transportation of chemotherapy drugs, leading to a lower sensitivity of chemotherapy than that of the tumor with relatively rich blood supply before chemotherapy. Although NIC has been proven to be a relatively stable indicators in tumor staging and detection of HER2 status (53, 54), its application and benefits are not entirely clear (55). Previous study revealed that NIC cannot serve as an independent predictive factor for lymph node metastasis in GC (51). Similarly, this study found that NIC was not statistically significant in univariate logistic analysis (*p* > 0.05); however, we still included NIC in the multivariate study and further confirmed it as a non-independent risk factor. Hence, future studies are prompted to discuss and validate the value of NIC in tumor prediction.

Tumor thickness plays an important role in predicting the therapeutic response of GC. Wang et al. revealed that tumor thickness ratio reduction was a good predictor of pathological complete response (pCR) after neoadjuvant chemotherapy (NAC) in patients with GC; however, tumor thickness before NAC was not helpful in predicting pCR (56). We also found that pre-treatment tumor thickness was not correlated with systemic chemotherapy response. Previous studies have suggested that the clinical staging of GC is closely related to the choice of treatment strategy and prognosis. At the same time, we have also demonstrated IC and clinical staging were significantly associated with systemic chemotherapy response, and an improvement of predictive power was observed when IC and clinical staging were added to the radiomics model.

Our study had some limitations. First, this is a pilot study using functional imaging radiomics to predict response to systemic chemotherapy. Although data from two centers were included, the sample size was still limited and lacked a validation group. However, the construction of our radiomics prediction model was based on features screened out using 10-fold cross-validation, ensuring optimal reliability and reproducibility. Furthermore, future collaboration with other dual-energy CT centers is underway to enlarge the sample size. Second, the current study gathered dual-center dual-energy data to increase the statistical power at the expense of increased variability of different manufacturers’ scanners; however, we used monochromatic images derived from the fast kilovolt peak-switching and dual-source acquisition paradigms to extract radiomics features to reduce the possible variability. Jacobsen et al. confirmed in a large phantom study that fast kilovolt peak-switching and dual source usually provided the most accurate monochromatic attenuation, and little difference existed in monochromatic error between the two scan protocols used in our study (57). Meanwhile, predictive model developed by current research achieved favorable performance, which further shows the good predictive value of the radiomics features based on different manufacturers’ dual-energy CT for chemotherapy response. Future research can attempt to use uniform dual-energy scanners and standardized imaging techniques to establish predictive model.

In conclusion, we developed a pre-treatment dual-energy CT–based radiomics nomogram for predicting clinical response to systemic chemotherapy in patients with AGC. Our preliminary results revealed that integrating multidimensional data including radiomics, clinical factors, and dual-energy parameter could benefit risk stratification, optimize candidate selection for systemic chemotherapy, and, finally, improve quality of life in patients with advanced gastric cancer.

## Data Availability Statement

The original contributions presented in the study are included in the article/**Supplementary Material**. Further inquiries can be directed to the corresponding author.

## Ethics Statement

The studies involving human participants were reviewed and approved by the medical ethical committee of Zhengzhou University and Ruijin Hospital. Written informed consent for participation was not required for this study in accordance with the national legislation and the institutional requirements.

## Author Contributions

Y-yL: manuscript preparation, literature research, and data analysis. HZ: manuscript preparation and literature research. LW: manuscript preparation and data collection. S-sL: data analysis. HL: data collection. H-jL: data analysis assistance. PL and JL: imaging analysis. P-jL: manuscript editing. J-bG: study conception and design, manuscript review, and guarantor of integrity of the entire study. All authors contributed to the article and approved the submitted version.

## Funding

Funding was provided by the National Natural Science Foundation of China (No.81971615) and the National Key Research and Develop Program of China (No. 2017YFC0112602).

## Conflict of Interest

Author S-sL was employed by the company Siemens Healthineers Ltd.

The remaining authors declare that the research was conducted in the absence of any commercial or financial relationships that could be construed as a potential conflict of interest.

## Publisher’s Note

All claims expressed in this article are solely those of the authors and do not necessarily represent those of their affiliated organizations, or those of the publisher, the editors and the reviewers. Any product that may be evaluated in this article, or claim that may be made by its manufacturer, is not guaranteed or endorsed by the publisher.
