# Supplementary material for: Predicting Response to Systemic Chemotherapy for Advanced Gastric Cancer Using Pre-Treatment Dual-Energy CT Radiomics: A Pilot Study
Source: Front Oncol. 2021 Sep 15;11:740732. doi: 10.3389/fonc.2021.740732 (PMC8480311; doi:10.3389/fonc.2021.740732)
Supplement: Supplementary file 1 [file DataSheet_1.docx]

**Appendix 1; Figure S1**


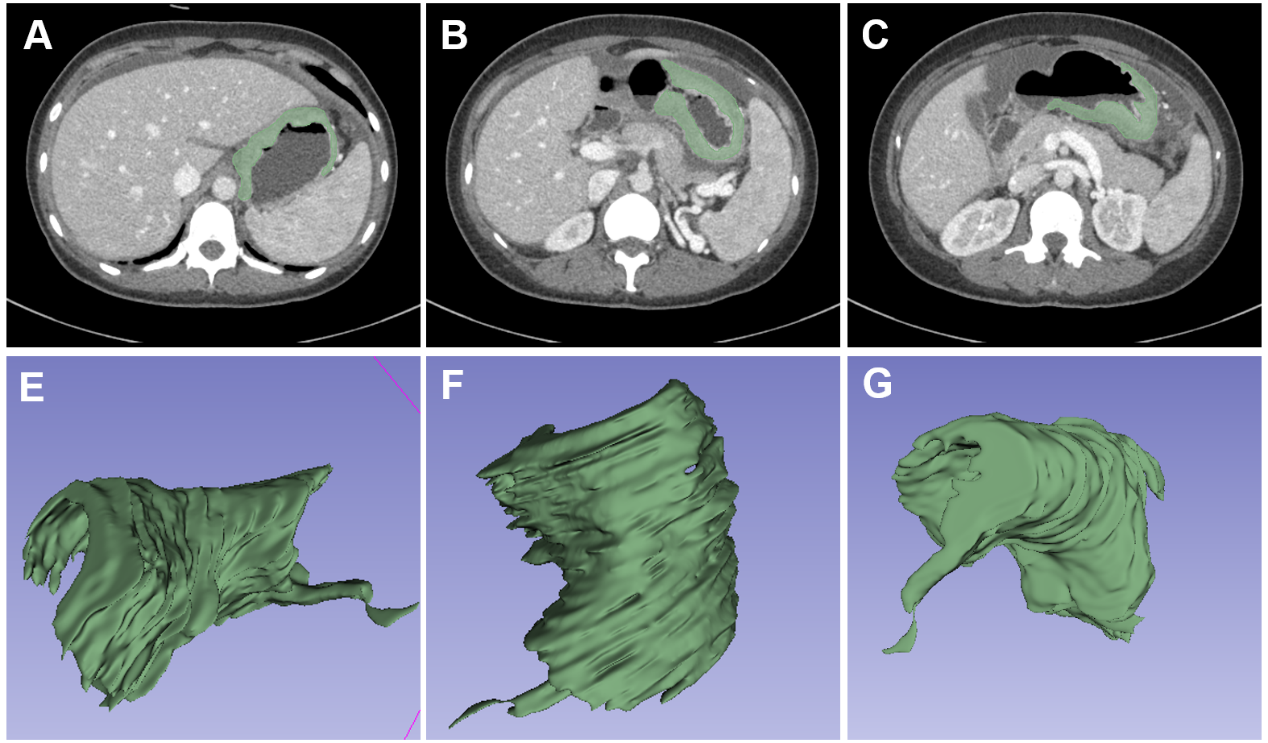


**Examples of manual segmentation of volumes of interest (VOIs).**

**(A-C)** showed the reference of manual segmentation for three VOIs on 70keV images (axial).  **(E-G)** 3D presentation of VOIs.

In addition，the VOIs automatically matched to the 40keV and 100keV images.

**Appendix 2. Tumor segmentation and feature extraction**

In each single-energy image set, there were 1691 radiomic features extracted for each patient, 110 of which were from the original image, 465 (93 x 5) of which were from the LoG-ﬁltered images, 744 (93 x 8) of which were from the wavelet-transformed images, and 372 (93 x 4) of which were from the non-linear intensity transforms. Table A1 shows the composition of all 1691 radiomic features.

**Table A1. Composition of the 1691 radiomic features**

|  | **Original** | **LoG-filtered** | **Non-linear transform** | **Wavelet** |
| --- | --- | --- | --- | --- |
|  | (n=1) | (n=5) | (n=4) | (n=8) |
| **Shape(n=17)** | 17x1 | - | - | - |
| **First order (n=18)** | 18x1 | 18x5 | 18x4 | 18x8 |
| **Texture (n=75)** | 75x1 | 75x5 | 75x4 | 75x8 |

**Appendix 3; Figure S2. Feature selection and radiomics model establishment**


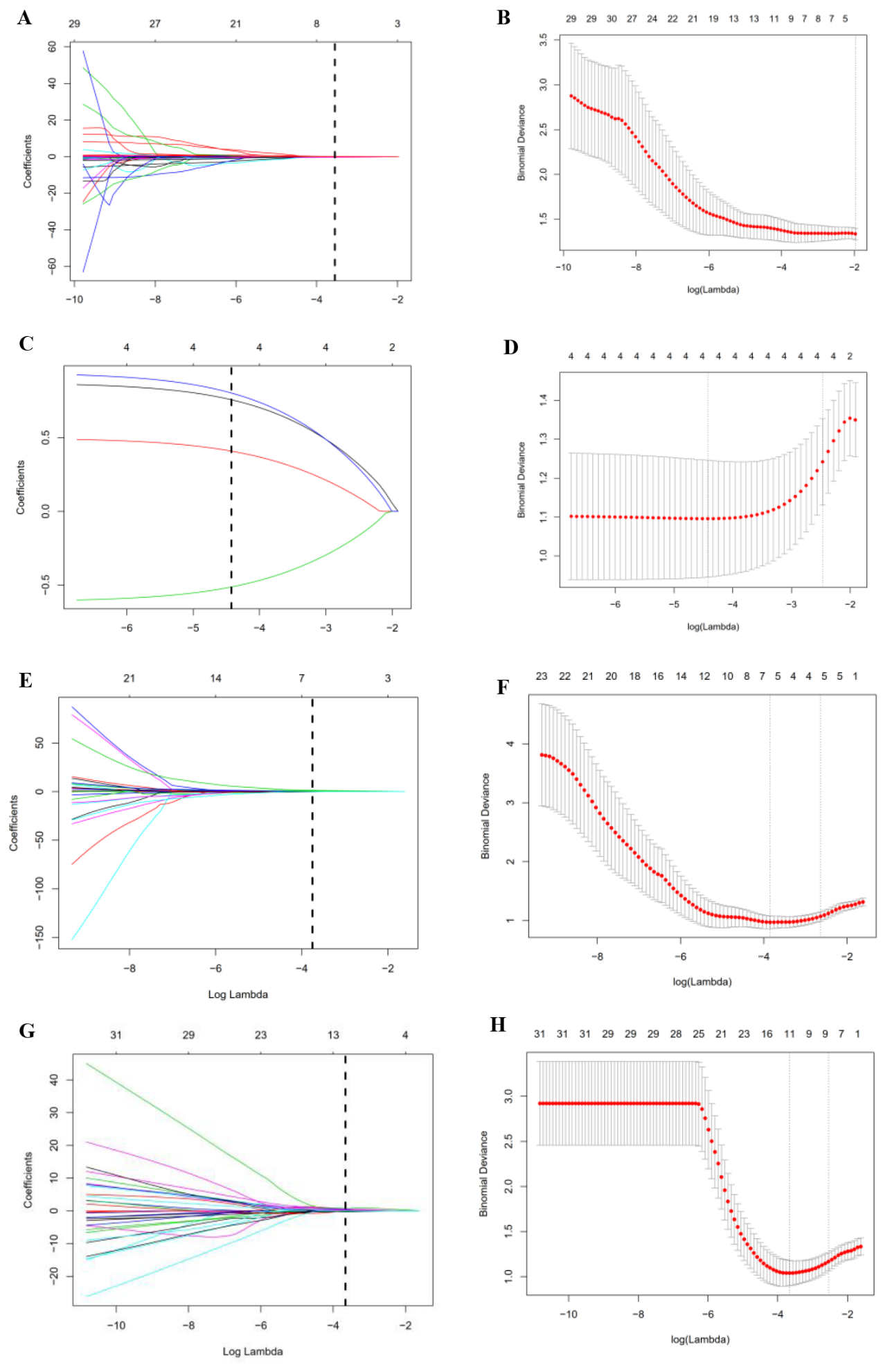


**FigureS2**. Process of LASSO regression of multi-energy radiomics feature selection.  **(A, C, E and G)** LASSO coefficient profiles of the radiomics features. A coefficient profile plot was produced against the log(λ) sequence, resulting in 8,4, 6 and 11 nonzero coefficients at the value selected by tenfold cross-validation in A，C，E and G, respectively. **(B，D, F and H)** Selection of tuning parameter **(λ).** That showed that the area under the curve (AUC) curve was plotted by the tuning parameter selection conducted by 10-fold cross-validation based on minimum deviance criterion. (A, B) 40keV features group, (C, D) 70keV features group, (E, F) 100keV features group, and (G, H) combined 40keV, 70keV and 100 keV feature group.

**Appendix 4; Table A2. Baseline Characteristics of All Patients.**

^a^ peritoneal, distant lymph node, adrenal gland, ovary; *N*=lymph node node, *IC*=iodine concentration,

| **Characteristics** | **NO.** | **%** |
| --- | --- | --- |
| Age (years)  Sex  Female  Male  ESCOG  PS 0  PS 1  Clinical Stage  III  IV  N stage  N0  N1  N2  N3  Metastatic sites  Absent  Liver  Lung  Other ^a^  Location  Upper  Middle  Lower  Diffuse  Borrmann type  I-II  III  IV  Thickness（cm）  IC (100μg/mL )  NIC | 54.6±13.8    21  48  40  29    22  47    11  27  16  15    29  14  2  24  30  12  17  10    10  35  24  2.40±0.84  22.84±3.64  0.105（0.09，0.137） | 30.4%  69.6%  58.0%  42.0%  31.9%  68.1%  15.9%  39.1%  23.1%  21.7%  42.0%  20.3%  2.9%  34.8%  43.5%  17.4%  24.7%  14.5%  14.5%  50.7%  34.8% |

*NIC*= normalized iodine concentration.

**Appendix 5.**  **Radiomics feature selection**

**TableA3.** Radiomics features contained in the single-energy models and their coefficients.

|  | **Feature** | **Coefficient** |
| --- | --- | --- |
| **40keV**  **70keV**  **100keV** | Intercept  squareroot_firstorder_Skewness_40  wavelet.HHH_glcm_MCC_40  wavelet.HHH_gldm_LargeDependenceHighGrayLevelEmphasis_40  wavelet.LHH_glszm_LargeAreaHighGrayLevelEmphasis_40  wavelet.LHL_glcm_Autocorrelation_40  wavelet.LHL_glrlm_ShortRunHighGrayLevelEmphasis_40  log.sigma.0.5.mm.3D_glcm_Imc1_40  log.sigma.1.5.mm.3D_glszm_GrayLevelVariance_40  Intercept  square_glszm_GrayLevelNonUniformity_70 square_glszm_GrayLevelNonUniformityNormalized_70 square_glszm_SmallAreaEmphasis_70  "log.sigma.2.5.mm.3D_glcm_Imc1_70  Intercept  squareroot_firstorder_Maximum_100  wavelet.HHH_glcm_ClusterShade_100  wavelet.HHL_glszm_GrayLevelNonUniformityNormalized_100  wavelet.LLL_firstorder_Maximum_100  log.sigma.1.5.mm.3D_firstorder_Skewness_100  log.sigma.2.5.mm.3D_glcm_Imc1_100 | -0.717987285040624  -0.131536581860485  -0.0996700212013053  -0.229988082775119 0.138990966630178  -0.000132098582811128 -0.0794532832349723  -0.157516434110668  -0.34692673592903  -0.795563855812507  0.756992051938729  0.408750976840541  -0.513100061631465 0.802816102812087  -0.877318492618349 0.513659189134672 0.504234077272568  0.706325564273858 0.0141412766170768  -0.160235271606764 1.33633524071 |

**TableA4.** Radiomics features contained in the multi-energy models and their coefficients.

|  | **Feature** | **Coefficient** |
| --- | --- | --- |
| **Full** | Intercept  squareroot_firstorder_Skewness_40  wavelet.LHH_glszm_LargeAreaHighGrayLevelEmphasis_40  square_glszm_GrayLevelNonUniformity_70  square_glszm_GrayLevelNonUniformityNormalized_70  square_glszm_SmallAreaEmphasis_70  log.sigma.2.5.mm.3D_glcm_Imc1_70  wavelet.HHH_glcm_ClusterShade_100  wavelet.HHL_glszm_GrayLevelNonUniformityNormalized_100 wavelet.LLL_firstorder_Maximum_100  log.sigma.1.5.mm.3D_firstorder_Skewness_100  log.sigma.2.5.mm.3D_glcm_Imc1_100 | -0.885673330764659  -0.292627376270898 0.204555676883388 0.0315605908970764 0.434953280531944  -0.191631729792989 0.579544423722352  0.445913064638508  0.507454033950876  0.413697280766966  -0.0787118032268702 0.708656775664434 |

Full: multi-energy
